# A Case of Common Carotid Artery Occlusion: A Unique Pattern of Occlusion and Lessons Learned

**DOI:** 10.3400/avd.cr.21-00080

**Published:** 2022-03-25

**Authors:** Thilina Gunawardena, Manujaya Godakandage, Sachith Abeywickrama, Balasubramaniyam Saseekaran, Rezni Cassim, Mandika Wijeyaratne

**Affiliations:** 1 Sir Peter Medawar Unit, Royal Liverpool University Hospital, Liverpool, the United Kingdom; 2 University Vascular Surgical Unit, National Hospital of Colombo, Colombo, Sri Lanka; 3 Department of Vascular Surgery, Worcester Acute Care Hospitals, Worcester, the United Kingdom; 4 Department of Renal Transplantation, Royal Free Hospital, London, the United Kingdom

**Keywords:** common carotid artery occlusion, internal carotid ligation, stroke

## Abstract

Common carotid artery occlusion (CCAO) is a rare cause of cerebrovascular events. For patients with neurological deficits due to emboli from an occluded common carotid artery, ligation of the ipsilateral internal carotid may appear as a simple therapeutic option, provided there is adequate collateral circulation. Here, we describe a patient with an unusual pattern of CCAO, who underwent ligation of the internal carotid artery after a successful test occlusion with a hypotensive challenge. He suffered a delayed stroke. This case report aims to highlight the unique pattern of CCAO in this patient and our experience with his management.

## Introduction

Common carotid artery occlusion (CCAO) accounts for 1%–5% of all cerebrovascular events.^1–3)^ The neurological symptoms seen in this condition are secondary to cerebral hypoperfusion or embolism. Unlike occlusion of the internal carotid artery (ICA), which has been studied extensively, literature on CCAO is sparse.^1)^ In this case report, we present a patient with left CCAO, who had a prior history of left-sided carotid endarterectomy (CEA). He was investigated for recurrent transient ischemic attacks (TIAs) after the CEA, and an unusual pattern of CCAO was identified. After a successful test occlusion (TO) of his left ICA with a hypotensive challenge, we performed an ICA ligation. Two days after the procedure, he developed an ischemic stroke and had to undergo an emergency axillary artery to ICA bypass.

## Case Report

**Figure 1:**
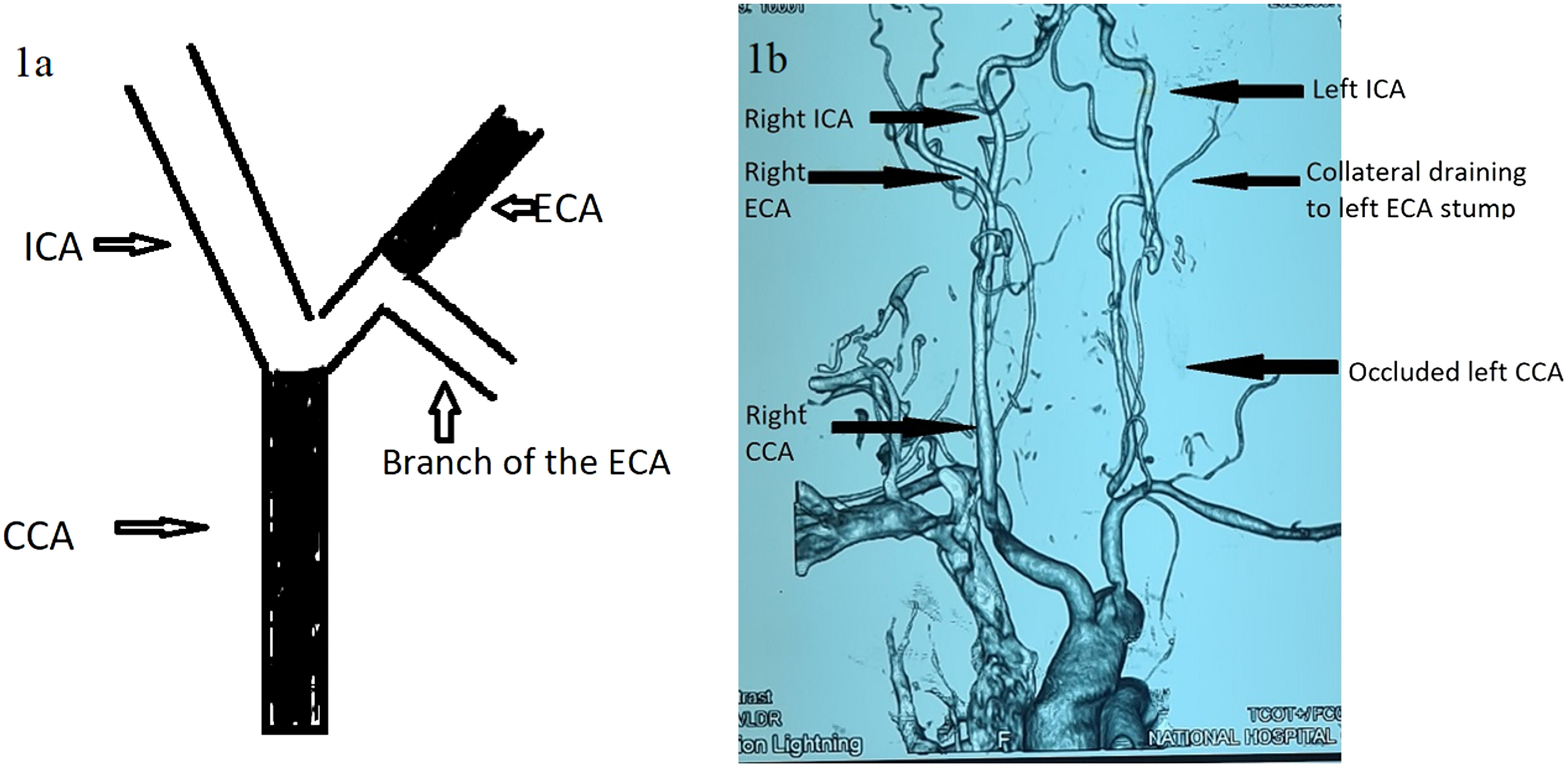
Fig. 1 (**a**) The pattern of common carotid artery occlusion in our patient, and (**b**) computed tomography angiogram of the carotid vessels.

A 57-year-old patient was investigated for two episodes of transient right upper limb and lower limb weakness. He was a heavy smoker and hypertensive. A non-contrast computed tomography (NCCT) scan of the brain excluded an intracranial hemorrhage (ICH) or an obvious infarction. Duplex ultrasound (DUS) showed a plaque causing 70% stenosis at the origin of the left ICA. The ipsilateral common carotid artery (CCA) and the contralateral carotid vessels were free from significant disease. Transthoracic echocardiogram excluded the heart as a possible source of emboli. The patient underwent left-sided CEA under general anesthesia with a shunt. The upper end of the plaque was tacked, and the arteriotomy was closed with a venous patch. The postoperative recovery was uneventful, and the patient was continued on dual antiplatelet therapy, atorvastatin, and antihypertensives. Six months following the surgery, the patient began to re-experience recurrent episodes of transient right arm and leg weakness. Repeat NCCT brain and the 2D echocardiogram were normal. The DUS showed complete occlusion of the left CCA. The arterial lumen was patent only at its bifurcation, and antegrade flow was seen in the patent left ICA. The left external carotid artery (ECA) was occluded just distal to its origin. Retrograde flow was noted in a branch draining to the origin of the external carotid (**Fig. 1a**). This vessel was responsible for maintaining the antegrade flow seen in the ICA. A computed tomography angiogram of the carotid vessels confirmed the patency pattern seen on the duplex (**Fig. 1b**).

**Figure 2:**
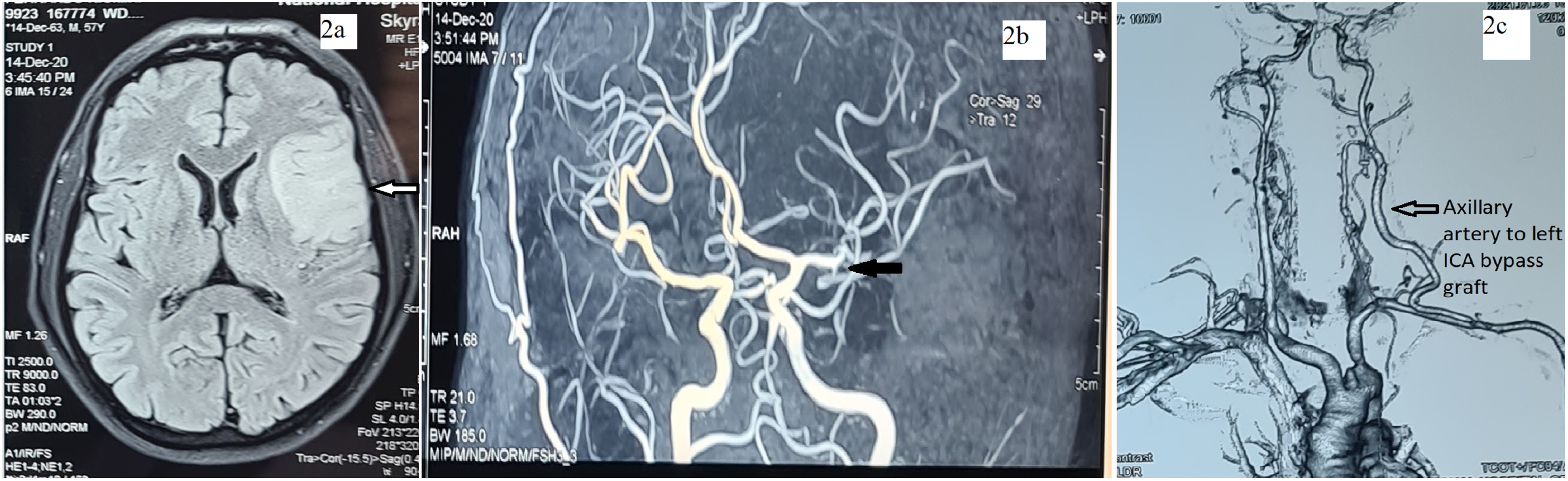
Fig. 2 (**a**) Magnetic resonance imaging showing the acute post-operative middle cerebral artery (MCA) territory infarction (arrow), (**b**) Magnetic resonance angiography of the occluded MCA (arrow), and (**c**) patent bypass graft on computed tomography angiogram (arrow).

We suspected embolism from the carotid stump as the root cause for the patient’s symptoms, and a decision was taken to ligate the internal carotid artery with or without a bypass, depending on the results of temporary ICA clamping. The surgery was done under local anesthesia with intra-arterial blood pressure monitoring. The left common, internal, and ECA control was taken. Because of scarring caused by the previous dissection, the retrograde feeder to the external carotid could not be specifically identified. The external carotid was clamped at its origin, and the systolic stump pressure of the internal carotid was taken, which was 44 mmHg. Despite keeping the clamps on for 20 min and a hypotensive challenge that lowered the mean arterial pressure (MAP) to 2/3rd of the patient’s baseline, there was no deterioration in the neurological status of the patient; thus, we went ahead with ICA ligation without a bypass. On postoperative day 2, the patient developed expressive aphasia that rapidly progressed to a right upper limb weakness. A repeat NCCT excluded an ICH. We suspected cerebral hypoperfusion or embolism from the ligated ICA stump, and immediately took him back to the operating theatre, he underwent immediate re-operation. The ligated ICA was opened up, and a fresh thrombus was noted immediately adjacent to the suture line. Brisk back-bleeding from the ICA was confirmed after removal of this thrombus, and a left axillary artery to ICA bypass was performed using the right greater saphenous vein. After recovery from anesthesia, an immediate improvement of the muscle power was noted; however, the aphasia persisted. A magnetic resonance imaging (MRI) scan of the brain with magnetic resonance angiography (MRA) that was done afterward showed an acute infarct due to embolic occlusion of the left middle cerebral artery (MCA) (**Figs. 2a** and **2b**).

After 3 months from the event, the patient’s aphasia has greatly improved with speech therapy. He is on best medical therapy, and there have been no further TIAs. The bypass remains patent on the surveillance CT angiogram (**Fig. 2c**). Informed, written consent was obtained from the patient before the collection of data and publication of the case report.

## Discussion

CCAO is a rare disease, and it accounts for 1%–5% of all cerebrovascular events.^1–3)^ Because of the rare nature of this condition, there is a paucity of suitable quality data with regard to its natural history, management, and treatment outcomes.^1)^ CCAO can present with a stroke, TIA, or features of cerebral hypoperfusion such as a drop attack.^3)^ Atherosclerosis is the leading cause of this pathology. The carotid bifurcation is a common location for atherosclerotic plaques, and retrograde extension of this disease along the CCA is possible.^3)^ Vasculitis, neck irradiation, fibromuscular dysplasia, neck trauma, cardiac embolism, dissection of the aortic arch or the CCA, and hypercoagulability are other rare causes of CCAO.^4)^ In our case, we suspect that intimal damage to the CCA from vascular clamp application or passage of the shunt during the previous CEA and progressive atherosclerosis may have led to the CCAO.

**Figure 3:**
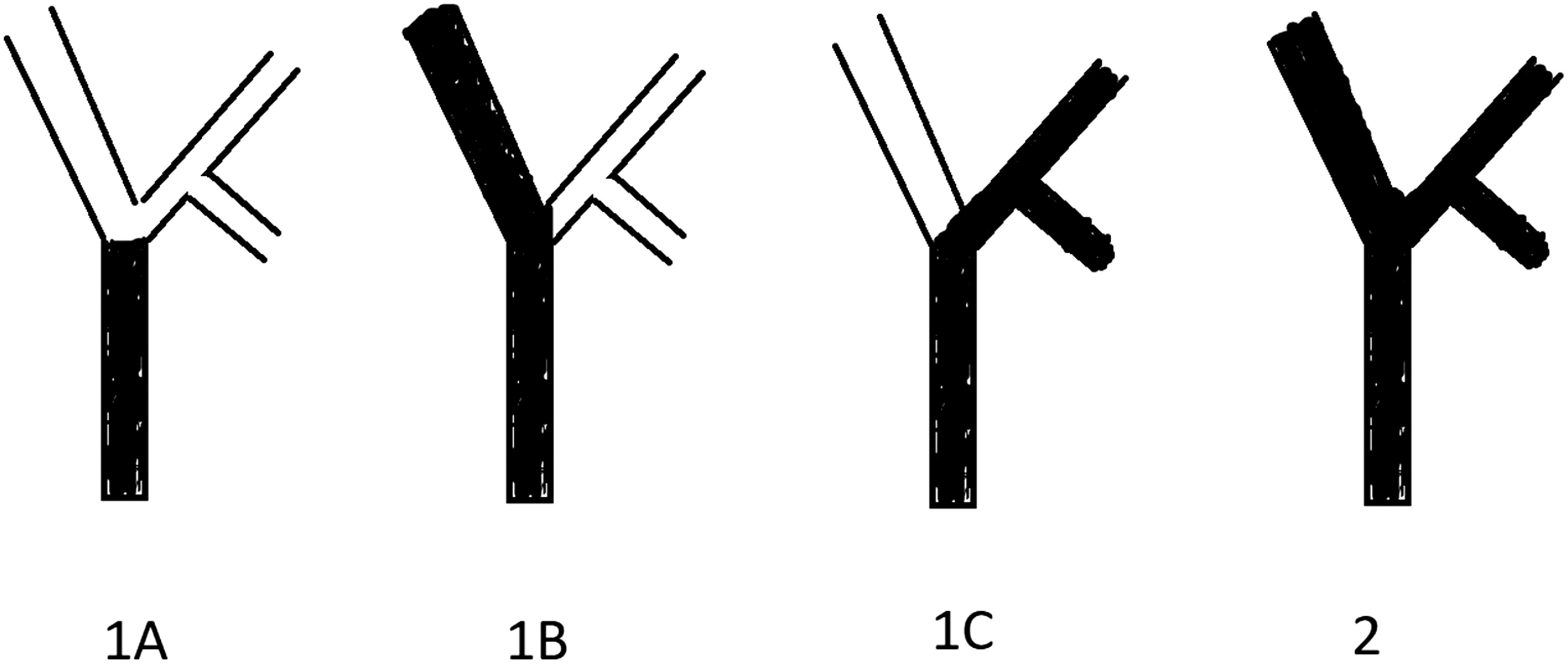
Fig. 3 Ryle’s classification of common carotid artery occlusion.

Rile described four patterns of CCAO (**Fig. 3**).^5)^ According to Klonaris et al., Type 1A was the commonest pattern, followed by Type 1B.^1)^ In the case series reported by Belczak et al., 2.5% of CCAOs were Type IC.^3)^ The pattern of obstruction seen in our patient has not been described before. He had an occluded CCA, and his ECA was occluded just beyond its origin. The ICA was completely patent. There was a branch with retrograde flow draining into the proximal stump of the ECA. This flow was continuing forward along the ICA. The pattern illustrates that even minor antegrade flow in the ICA with an occluded CCA can cause cerebrovascular events due to emboli.

The ultimate goal in treating CCAO is stroke prevention. For asymptomatic patients with CCAO, medical treatment alone is an acceptable strategy.^1,3)^ Patients who have symptoms due to hypoperfusion of the brain will require some form of revascularization. For this purpose, bypass procedures, transposition of the CCA, CCA endarterectomy, and endovascular re-canalization of the occluded CCA are therapeutic options.^1)^

Ligation of the ICA has been utilized to manage head and neck tumors, neck trauma, and aneurysmal disease of the carotid arteries.^6,7)^ For patients with symptoms attributable to emboli originating from occluded CCA, ligation of the ICA has not been considered a first-line treatment strategy. Provided there is adequate collateral perfusion through the circle of Willis, this presents as a simple, attractive therapeutic option that can be performed under local anesthesia; however, cerebral hypoperfusion due to CCAO should be definitively excluded, and the adequacy of the collateral circulation should be confirmed before attempting ICA ligation.

Ligation of the ICA without confirming the adequacy of collateral circulation is associated with an unacceptable risk of stroke that can be as high as 49%. Even after a successful TO of the ICA, 5%–20% will experience a stroke. Objective measurement of cerebral blood flow at the time of TO using techniques such as single-photon emission computed tomography may further reduce the risk of stroke to 5%–8%.^7)^

ICA balloon occlusion combined with a hypotensive challenge has been described as a reliable method for confirming the adequacy of collateral perfusion before ICA ligation. As per Standard et al., after such a negative test, the risk of stroke due to cerebral hypoperfusion was negligible.^7)^ In the case of our patient, we used a modification of this technique before embarking on ICA ligation. We performed an awake TO of the ICA with a hypotensive challenge. As there was no neurological deterioration during this test, we concluded that his collateral circulation would compensate after the left ICA was ligated. Additionally, the test eliminated cerebral hypoperfusion as the likely cause for the patient’s symptoms. The lowering of the MAP below the normal range in the form of a hypotensive challenge is important to allow for fluctuations of blood pressure that occur outside controlled surgical conditions.

Cerebral hypoperfusion and thrombo-emboli originating in the stagnant blood within the ligated ICA stump have been described as causes for stroke after ICA ligation. The former will manifest with an immediate post-operative stroke, whereas the latter will be responsible for cerebral deficits 12 h to 7 days after the procedure.^8)^ Embolism was the likely mechanism for the delayed stroke observed in our patient, which was proven subsequently by MRA.

As a solution for this delayed onset stroke, we undertook an emergency left axillary artery to left ICA saphenous vein bypass. The ipsilateral subclavian artery is an alternative inflow vessel for revascularization of an occluded CCA. Exposing the axillary artery is comparatively easy, and it does not have a close relationship with the brachial plexus and the thoracic duct as the subclavian artery.^9)^ Conversely, some favor the subclavian artery as an inflow because the bypass can be done in a single operative field with the use of a shorter conduit. In our case, the axillary artery was chosen primarily because we are more familiar with its exposure, and we felt that the situation demanded expeditious surgery. After the revascularization of the ICA, the patient’s motor weakness improved, probably because of reperfusion of the ischemic penumbra. The aphasia slowly recovered with aggressive speech therapy.

## Conclusion

In retrospect, we believe that the patient should have been treated with ICA ligation combined with a bypass in the first instance. Although an intra-operative awake TO of the ICA with a hypotensive challenge may be tolerated, it will not guarantee that the patient will be free of stroke due to hypoperfusion, as blood pressure outside controlled circumstances can be quite variable. Additionally, stagnant blood within the ligated stump of the ICA can be a new source of embolism.
